# Clinical significance of Vitamin-D and other bone turnover markers on bone mineral density in patients with gestational diabetes mellitus

**DOI:** 10.12669/pjms.38.1.4461

**Published:** 2022

**Authors:** Jingjing Ma, Lulu Han, Xue Zhou, Zhihong Li

**Affiliations:** 1 Jingjing Ma, Department of Endocrinology, Baoding First Central Hospital, Baoding, Hebei, 071000, China; 2 Lulu Han, Department of Endocrinology, Baoding First Central Hospital, Baoding, Hebei, 071000, China; 3 Xue Zhou, Department of Endocrinology, Baoding First Central Hospital, Baoding, Hebei, 071000, China; 4 Zhihong Li, Department of Endocrinology, Baoding First Central Hospital, Baoding, Hebei, 071000, China

**Keywords:** Gestational diabetes mellitus, Vitamin-D, Bone turnover markers, Bone mass density

## Abstract

**Objectives:**

To perform a correlation analysis of serum 25-hydroxyVitamin-D[25-OH-D], bone turnover markers (BTMs), and bone mineral density (BMD) in patients with gestational diabetes mellitus (GDM) during mid- and late pregnancy and state the significance of these factors for guiding clinical prevention and control of GDM.

**Methods:**

This study involved 100 pregnant women with singleton pregnancies who visited our obstetrics and gynecology department, Baoding First Central Hospital, during January 2019 and December 2020. All participants had received more than five prenatal checkups and were assigned to a GDM group and a normal group according to the presence of GDM during the mid-pregnancy period. Serum 25-OH-D, BMD, and bone turnover markers (BTMs) were measured to analyze the differences between the two groups and observe possible correlations among these factors.

**Results:**

According to the examination results, GDM occurred in 31 (31%) participants, and the rest 69 (69%) were free of GDM during mid-pregnancy. No significant differences were observed between the two groups in basic clinical data and the serum levels of high-density lipoprotein cholesterol (HDL-C), low-density lipoprotein cholesterol (LDL-C), and triglyceride (TG) (*p*>0.05), whereas the fasting blood glucose (FBG) level in the GDM group was significantly higher than in the normal group (*p*<0.05). The serum 25-OH-D and BMD levels in the GDM group were lower than in the normal group, whereas the bone alkaline phosphatase (BALP), osteocalcin (OC), procollagen type I propeptide (PINP), and beta-isomerized C-terminal telopeptide (β-CTx) levels in the GDM group were significantly higher than in the normal group, with the differences showing statistical significance (*p*<0.05, respectively). The results of Pearson’s correlation analysis revealed that serum 25-OH-D was positively correlated with BMD (r =0.183, P <0.05) and negatively correlated with such BTMs as BALP, OC, PINP, and β-CTx (r =-0.255, -0.369, -0.204, -0.610; *p*<0.05).

**Conclusion:**

During mid and late pregnancy, GDM patients are prone to Vitamin-D deficiency, which has an adverse effect on bone turnover, BMD, and even the health of the mother and the development of the fetus. Therefore, routine screening for Vitamin-D deficiency is recommended throughout pregnancy.

## INTRODUCTION

Pregnancy is a special physiological condition associated with complex physiological changes. Pregnant women are faced with considerable changes in their blood, circulation, and immune systems and have unique nutritional needs to support healthy development of the fetus. Research shows that most pregnant women have varied degrees of 25-hydroxyVitamin-D (25-OH-D) deficiency during mid- and late pregnancy.^1^ As a key player in bone mineral metabolism, 25-OH-D not only modulates calcium and phosphorus metabolism and the cardiovascular system but also supports muscle strength. Reportedly, Vitamin-D deficiency is a common clinical manifestation in patients with diabetes mellitus (DM), and in a pregnant woman, this may affect the stomach and the placenta, interfering with the healthy bone development of the fetus.^2^ Clinical knowledge of the correlation between serum 25-OH-D and bone turnover in mid- and late pregnancy is so far very limited. Gestational diabetes mellitus (GDM) is a common medical complication of pregnancy, which predisposes pregnant women to 25-OH-D deficiency and affects their bone turnover and bone mineral density (BMD).^3^ Focusing on pregnant women with GDM, this study measured serum 25-OH-D, BTMs, and BMD and analyzed the effects of serum 25-OH-D and relevant BTMs on BMD and the correlations of these factors, aiming to facilitate clinical diagnosis and treatment of GDM with practical guidance.

## METHODS

### Clinical Data:

This study included 100 pregnant women who first visited our bstetrics and gynecology department, Baoding First Central Hospital, during January 2019 and December 2020. and received prenatal checkups at regular intervals.

### Ethical Approval:

The study was approved by the Institutional Ethics Committee of Baoding First Central Hospital (No. 2019064; date: November 18, 2019), and written informed consent was obtained from all participants

### Inclusion criteria:

• Gestational age ≥20 weeks;

• 20-35 years old;

• Ssingleton pregnancy

• Prenatal checkups >5 visits;

• Informed consent for this study and a high degree of cooperation throughout the study.

### Exclusion criteria:

• Presence of comorbid conditions, such as gestational hypertension, preeclampsia, and heart disease;
• Complicated with thyroid or parathyroid disorders or pre gestational diabetes;
• Preexisting mental disorders;
• With a history of infectious diseases;
• Early withdrawal.

The participants were 22 to 34 years old, with the mean age of (27.92 ±3.34) years; the gestational age ranged from 20 to 25 weeks, with the mean gestational age of (23.15 ±2.73) weeks; the body weight fell between 57.22 and 77.43 kg, with the mean body weight of (64.22 ±7.12) kg; there were 62 primiparous and 38 multiparous mothers. This study was approved by the hospital’s medical ethics committee.

Venous blood (5 mL) was drawn from each participant on an empty stomach in the morning, and centrifugation was performed to collect the supernatant. A fully automatic biochemistry analyzer was utilized to measure the fasting blood glucose (FBG), high density lipoprotein cholesterol (HDL-C), low density lipoprotein cholesterol (LDL-C), and triglyceride (TG) levels. A chemiluminescence immunoassay (CLIA) analyzer (Nanjing Norman, China) was used to determine the serum 25-OH-D and OC levels. The ELISA was performed based on a biochemical analysis system (Roche, Germany) to detect the levels of PINP and β-CTx. A CM-200 ultrasound bone densitometer (Furuno, Japan) was employed to measure the BMD of the right heel and recorded the Z-score. All tests were performed by a clinician and an experienced physician.

### Screening for GDM:

A glucose tolerance test (GTT) was taken to diagnose or exclude GDM. In addition to FBG, the 1 hours and 2 hours postprandial blood glucose (1hPBG and 2hPBG) levels were also measured, and one was confirmed to have GDM if she met any of the following criteria: FBG >5.1 mmol/L, 1hPBG >10.0 mmol/L, and 2hPBG >8.5 mmol/L.

### Statistical Analysis:

The software SPSS22.0 was used for data processing. Measurement data were expressed in the form of “mean ±standard deviation (X̄±s)”, with intergroup comparisons being examined by the t-test. Enumeration data were denoted by number of cases (n) and percentage (n%), and intergroup comparisons were examined by the χ2 test. Pearson’s r was used for correlation analysis. P <0.05 was considered statistically significant.

## RESULTS

According to the GTT results and clinical manifestations, 31 (31%) pregnant women were confirmed to have GDM, and the rest 69 (69%) participants were free of GDM. The results of comparative analysis showed that the two groups had no statistically significant differences in the serum levels of HDL-C, LDL-C, and TG (*p*>0.05, respectively), whereas the FBG level in the GDM group was significantly higher than in the normal group (*p*<0.05). Table-I.

**Table I:** Comparison of basic clinical data between the GDM and normal groups.

Item		GDM Group (n =31)	Normal Group (n =69)	χ2/t	*p*
Gestational age (x̅ ±s, wks)		22.29 ±2.42	23.13 ±2.38	0.522	0.107
Parity (n, %)	Primip.	19 (61.29)	43 (62.32)	0.214	0.115
	Multip.	12 (38.71)	26 (37.68)		
Body weight (x̅ ±s, kg)		65.23 ±6.42	63.18 ±7.26	0.410	0.238
Age (x̅ ±s, yrs)		27.85 ±3.23	27.26 ±3.50	0.633	0.084
FBG (x̅ ±s, mmol/L)		5.79 ±0.62	4.64 ±0.53	4.063	0.010
HDL-C (x̅ ±s, mmol/L)		1.31 ±0.24	1.28 ±0.30	0.609	0.068
LDL-C (x̅ ±s, mmol/L)		1.56 ±0.29	1.42 ±0.18	0.957	0.074
TG (x̅ ±s, mmol/L)		2.24 ±0.38	2.17 ±0.40	0.152	0.061

The BMD and BTM test results revealed that the GDM group had lower levels of serum 25-OH-D and BMD, but higher levels of BALP, OC, PINP, and β-CTx compared with the normal group, and the differences were statistically significant (*p*<0.05, respectively). Table-II.

**Table II:** Comparison of BMD and BTMs between the GDM and normal groups.

Group	Cases	25-OH-D (ng/mL)	BMD (Z-score)	BALP (μg/L)	OC (ng/mL)	PINP (ng/mL)	β-CTx (ng/mL)
GDM Group	31	24.90 ±3.32	0.91 ±0.24	4.81 ±1.22	16.97 ±7.04	50.22 ±18.06	0.49 ±0.21
Normal group	69	39.58 ±5.74	1.12 ±0.30	3.92 ±1.04	14.08 ±5.81	43.60 ±17.25	0.38 ±0.27
t		14.052	4.063	6.224	10.074	15.695	3.015
p		0.000	0.001	0.001	0.012	0.000	0.010

Analysis of correlations of serum 25-OH-D with BMD and serum BTMs in GDM patients: Pearson’s correlation analysis was conducted to investigate the correlations of serum 25-OH-D with BMD and serum BTMs in GDM patients, and the results revealed that serum 25-OH-D was positively correlated with BMD (r =0.183, p<0.05) and negatively correlated with such BTMs as BALP, OC, PINP, and β-CTx (r =-0.255, -0.369, -0.204, -0.610; p<0.05, respectively). Table-III.

**Table III:** Correlations of serum 25-OH-D with BMD and serum BTMs in GDM patients.

Indicator	Serum 25-OH-D	

	r	p
BMD	0.183	0.001
BALP	-0.255	0.000
OC	-0.369	0.000
PINP	-0.204	0.001
β-CTx	-0.610	0.000

## DISCUSSION

Clinical studies have shown that bone mass is reduced in pregnant women with GDM compared with their healthy counterparts, and thus GDM is considered a major risk factor for osteoporosis.^4^,^5^ It has been found that bone mass loss appears to be associated with changes in hormones.^6^ In a pregnant woman, the estrogen level increases sharply as the placenta develops, and as a critical bone resorption inhibitor, estrogen interferes with the remodeling of bone tissue, increasing the risk of bone metabolism disorders in the mother and abnormal bone development in the fetus and adversely affecting the pregnancy outcome. Vitamin-D is a fat-soluble steroid hormone, and serum 25-OH-D is the major circulating form of Vitamin-D in the body, which not only plays a fundamental role in supporting human bone health but potentiates the development and progression of non-skeletal diseases.^7^ Despite all that, there is a lack of clinical studies focusing on the expression of Vitamin-D, BMD, and BTMs, as well as correlations among these factors, in pregnant women with GDM.

It is suggested that 25-OH-D provides structural and functional support for pancreatic β cells and thus can be used for modulating insulin resistance and alleviating relevant immune responses.^8^ A reduction in serum 25-OH-D can trigger a shutdown of the Ca^+^ pathway on the pancreatic β-cell membrane, contributing to abnormal production and release of insulin by islet cells and worsening of insulin resistance. The mid- and late pregnancy period involves fetal development and fast growth and calcification of bones, which means an increased need for Vitamin-D. Since the pregnant mother is the major source of Vitamin-D for the fetus, Vitamin-D deficiency without timely supplementation may affect the healthy growth and development of the fetus.^9^ It is reported in a international study^10^ that the occurrence of GDM is somewhat associated with Vitamin-D deficiency, which is probably attributed to the worsening of insulin resistance.

To make an objective evaluation of the correlations of serum 25-OH-D with BTMs and BMD during mid- and late pregnancy, this study presented a comparative analysis by measuring the serum 25-OH-D, BTMs, and BMD levels in GDM patients and healthy individuals during mid- and late pregnancy. The results of this study showed that the differences between the two groups in the HDL-C, LDL-C, and TG levels were not statistically significant, and the GDM patients had a significantly higher level of FBG compared with the healthy individuals during mid- and late pregnancy, which were consistent with the diagnostic conclusions. Besides, the measured results also revealed that compared with the normal group, the GDM group exhibited a marked reduction in the serum 25-OH-D and BMD levels and a substantial increase in the BALP, OC, PINP, and β-CTx levels, conforming with the findings reported in other studies.^11^,^12^ This indicates that in pregnant women with GDM, increased blood glucose is associated with the decreased expression of 25-OH-D, which reduces BMD and leads to bone metabolism disorders. Osteocalcin (OC) is a polypeptide hormone and noncollagenous protein that plays a regulatory role in Ca, P, and glucose metabolism. Numerous experiments have proved that OC can promote pancreatic β-cell growth, elevate the expression and secretion levels of insulin and regulate body fat.^13^,^14^ GDM can be viewed as an early sign of Type-2 diabetes mellitus (T2DM). The OC level increases to promote the release of insulin from pancreatic β cells as compensation for insulin resistance during pregnancy; however, no sufficient amounts of insulin are secreted because of a relative deficiency of pancreatic β cells, regardless of the increase in the OC level.^15^-^17^ It is suggested that the OC level rises as bone resorption markers increase, which promotes the occurrence GDM and induces bone mass loss, contributing to a lower BMD.^18^ BALP, PINP, and β-CTx are three vital BTMs mainly produced by and released from bones. A reduction in the BMD level can promote the release of these BTMs; otherwise, the serum levels of these BTMs reduce when BMD falls within the normal range.^19^,^20^ From the results of the correlation analysis, it was found that serum 25-OH-D has a positive correlation with BMD (*p*<0.05) and negative correlations with BALP, OC, PINP, and β-CTx (*p*<0.05, respectively). This indicates that during mid- and late pregnancy, GDM patients are prone to Vitamin-D deficiency and bone mass loss.

### Limitations and Recommendations of the study:

Nevertheless, deficiencies are still visible in this study: small sample size, short follow-up time, and no more accurate classification study on patients with Gestational Diabetes Mellitus. In addition, only patients with mild to moderate Gestational Diabetes Mellitus are selected as subjects to ensure the effectiveness of the study. Based on this, relevant countermeasures are being carried out to actively enrich the sample content, further extend the time of follow-up, and classify different types of Gestational Diabetes more precisely and further include severe patients with Gestational Diabetes Mellitus in the study.

## CONCLUSIONS

Compared with healthy pregnant women, GDM patients are exposed to a higher risk of Vitamin-D deficiency during mid- and late pregnancy, which may affect bone turnover and BMD and undermine the health of both the mother and the fetus. Therefore, surveillance and monitoring of serum 25-OH-D, BMD, and BTMs should be enhanced to ensure good pregnancy outcomes among women with GDM.

### Authors’ Contributions:

**JM & LH:** Designed this study and prepared this manuscript, are responsible and accountable for the accuracy, integrity of the work.

**XZ:** Collected and analyzed clinical data.

**ZL:** Significantly revised this manuscript.
